# Healthcare Expenditures Associated with Heart Failure in Saudi Arabia: A Cost of Illness Study

**DOI:** 10.3390/healthcare9080988

**Published:** 2021-08-04

**Authors:** Ahmed Alghamdi, Eman Algarni, Bander Balkhi, Abdulaziz Altowaijri, Abdulaziz Alhossan

**Affiliations:** 1 Department of Clinical Pharmacy, College of Pharmacy, King Saud University, Riyadh 11451, Saudi Arabia; BBalkhi@ksu.edu.sa (B.B.); alhossan@ksu.edu.sa (A.A.); 2 Prince Sultan Cardiac Center, Prince Sultan Military Medical City, Riyadh 12233, Saudi Arabia; eman.algarni@gmail.com; 3 Program for Health Assurance and Purchasing, Vision Realization Office, Ministry of Health, Riyadh 13315, Saudi Arabia; abdulaziz.altowaijri@gmail.com

**Keywords:** heart failure, healthcare expenditures, cost of illness

## Abstract

Heart failure (HF) is considered to be a global health problem that generates a significant economic burden. Despite the growing prevalence in Saudi Arabia, the economic burden of HF is not well studied. The aim of this study was to estimate the health care expenditures associated with HF in Saudi Arabia from a social perspective. We conducted a multicenter cost of illness (COI) study in two large governmental centers in Riyadh, Saudi Arabia using 369 HF patients. A COI model was developed in order to estimate the direct medical costs associated with HF. The indirect costs of HF were estimated based on a human capital approach. Descriptive and inferential statistics were analyzed. The direct medical cost per HF patient was $9563. Hospitalization costs were the major driver in total spending, followed by medication and diagnostics costs. The cost significantly increased in line with the disease progression, ranging from $3671 in class I to $16,447 in class IV. The indirect costs per working HF patient were $4628 due to absenteeism, and $6388 due to presenteeism. The economic burden of HF is significantly high in Saudi Arabia. Decision makers need to focus on allocating resources towards strategies that prevent frequent hospitalizations and improve HF management and patient outcomes in order to lower the growing economic burden.

## 1. Introduction

Heart failure (HF) is a complicated progressive disease that occurs when the heart fails to provide sufficient blood to the body in order to meet its metabolic demands [1,2]. The incidence and prevalence of HF have increased dramatically over the past decade, especially among older people. Globally, HF affects more than 64 million individuals and the prevalence among adults in developed countries was reported to be approximately 4.2%, with 11.8% of individuals aged 65 years or above [3,4,5].

HF is considered to be a leading cause of mortality, since approximately 40.2% of patients die within 2.5 years of their follow-up [6]. Furthermore, HF is associated with a significant clinical burden. Approximately 30–40% of patients have a history of frequent hospitalization, and two-thirds are readmitted within a year of their diagnoses [7,8,9]. In addition, HF has a significant negative impact on patients’ physical, mental and social well-being, leading to a deterioration in their quality of life [10].

In Saudi Arabia, the estimated number of patients diagnosed with HF was 455,222 in 2012, with approximately 32,200 new patients being projected to be diagnosed annually [11]. The mean age of patients diagnosed with chronic and acute HF ranged between 57 and 60 years, which is lower than that of many developed countries. In addition, the majority of patients exhibited moderate to severe left ventricular dysfunction [12,13].

Despite developments in the management of HF, the disease is expected to impose a heavy economic burden on healthcare systems due to its growing prevalence and the increased consumption of healthcare resources by HF patients. Globally, the total annual cost of treating HF patients was estimated at $108 billion, approximately 60% of which was related to direct medical costs [14,15]. In the United States of America (USA), the total cost of treating HF patients was $43 billion in 2020 and is expected to reach $69.7 billion by 2030. Elsewhere, the total cost was $6.8 billion in Brazil, $752.8 million in South Korea, and $103.6 million in Lebanon, while the treatment of HF consumed approximately 2% of the healthcare budget of both the United Kingdom (UK) and Sweden [16,17,18,19,20,21].

The majority of the economic burden of HF is attributed to direct medical costs, especially hospitalization costs. These account for 44–96% of the total healthcare expenditure. The median cost of treating each patient hospitalized with HF ranges between $13,418 and $15,879. This figure is expected to increase with comorbidities and the advanced stages of HF [22,23]. Furthermore, indirect costs incurred, such as loss of productivity due to HF morbidity and mortality, are considered to be a significant contributor to the total cost of treatment. It was estimated that approximately 24–40% of HF costs resulted from indirect costs due to absenteeism, presenteeism and informal care [15,24].

Although HF imposes a heavy economic burden on the healthcare systems and societies of various countries, evidence of the economic burden of HF in Saudi Arabia is limited. In 2018, a study targeting healthcare experts estimated the annual costs attributed to the treatment of HF in four Middle Eastern countries, including Saudi Arabia. The estimated costs were primarily based on experts’ opinions. In addition, the study only considered the payer’s perspective and did not estimate the indirect costs [25]. Another study conducted from the payer’s perspective focused only on the direct costs of treating HF patients with preserved ejection fraction (EF), rather than general HF patients [26].

Given the limitations of existing studies in evaluating the economic burden of HF in Saudi Arabia using real world data, this study sought to estimate the annual cost of HF treatment, considering both direct and indirect costs over a one-year follow-up of patients diagnosed with HF and seeking to understand the factors that drive such costs.

## 2. Methods

### 2.1. Design and Population

We conducted a prospective prevalence-based, multi-center cost of illness (COI) study at the Prince Sultan Cardiac Center (PSCC) and King Saud University Medical City (KSUMC) in Riyadh, Saudi Arabia. The study was conducted from a social perspective to include the estimations of direct and indirect medical costs. In order to estimate the economic burden and resources consumed, we developed a COI model based on the cost of the individual units of service performed. Using electronic medical records (EMR), we identified patients diagnosed with HF aged 18 years and above based on current HF guidelines and followed them for one year, from July 2019 to July 2020.

### 2.2. Data Collection

#### 2.2.1. Demographics and Disease Characteristics

We collected the patients’ demographics and the disease characteristics of patients diagnosed with HF and categorized the patients into four categories based on their inability to participate in physical activities, using the New York Heart Association (NYHA) classification [27]. In addition, we used the left ventricular ejection fraction (LVEF), a tool used to define the severity of HF based on ejection fraction (EF), in order to classify patients into two categories: those with reduced EF (HFrEF) (defined as EF ≤ 40%) and those with preserved EF (HFpEF) (defined as EF ≥ 50%) [28].

#### 2.2.2. Direct Costs

We adopted a micro-costing, bottom-up approach in order to estimate the cost of the consumption of healthcare resources directly related to the treatment of HF. First, we measured and quantified healthcare resources or interventions used by each patient during the period of study. We then estimated the unit costs for such interventions. To do so, we calculated the total direct cost by multiplying the unit cost with the quantity consumed and thus estimated the overall direct cost per patient by the end of the year.

We obtained healthcare services cost data, including the costs of hospitalization, conducting procedures, medications, diagnostics tests, lab tests, outpatient visits, emergency visits, counselling, and education services provided by different specialists, from the PSCC and KSUMC business centers. To estimate the overall direct cost of these services per patient, we used a weighted average for both institutional costs in order to decrease discrepancy between the estimated costs and to enhance the generalizability of the results. All cost calculations were based on 2020 prices.

#### 2.2.3. Indirect Costs

Indirect cost refers to the costs incurred by patients from the loss of their productivity as a result of being affected by the disease [29]. To estimate the indirect cost associated with HF, we used a human capital method to estimate the costs resulting from a loss in productivity among the patients who were working in the labor market. In this study, we defined loss in productivity as the absenteeism/presenteeism of the patient. We calculated absenteeism by estimating, in hours, the time lost by patients or the time provided by caregivers in HF management. We also calculated presenteeism by estimating, in hours, the reduction in productivity of patients because of HF. To estimate the total time lost for each category, a validated self-reported questionnaire was provided to the patients on every visit. The questionnaire determined the number of work hours lost by the patients every month because of inactivity due to HF. Thereafter, we calculated the total number of hours lost per month and per year and estimated the loss in productivity. Here, we considered the loss in income associated with loss in productivity as the indirect cost and estimated it based on the average wage per hour for actively working patients using the gross domestic product (GDP) per capita [30,31]. For this study, we collected the GDP per capita of Saudi Arabia for the year 2019 from the World Bank database and used it as an index for the estimation of indirect costs [32]. The calculations of all the costs were presented in the United States Dollar (1 USD = 3.75 Saudi Riyal).

### 2.3. Statistical Analysis

We performed descriptive statistics, including the calculation of means, standard deviation (SD), and frequencies. Additionally, we used inferential statistics as needed, including t-test and the analysis of variance (ANOVA) tests in order to compare the different HF groups. We also performed a regression analysis in order to analyze the association between the estimated costs and different variables, such as age, gender, EF, and comorbidities. All *p*-values less than 0.05 were considered statistically significant. We conducted statistical analyses using the Statistical Package for the Social Sciences (SPSS) version 21 (SPSS Inc., Chicago, IL, USA).

## 3. Results

A total of 369 patients diagnosed with HF were included in the study. Patients’ demographics and disease characteristics are summarized in Table 1. Approximately two-thirds of the studied population were males (64%), and the mean age (SD) was 53 (15) years. Of the study population, only 27% were employed, with the majority either retirees or unemployed. The majority of the patients were non-smokers (76%), and 65% of them were from the central region in Saudi Arabia. Approximately 46% of the patients had a disease duration of one to five years, and 71% had experienced one to three comorbidities. Among all the comorbidities that were present in the patients, diabetes, hypertension, and ischemic heart disease were the most commonly reported. According to the NYHA classification, 29% of the patients were classified as class I, 38% were classified as class II, 23% were classified as class III, and only 10% were classified as class IV. The majority of the patients were classified as HFpEF, with a mean EF (SD) of 59 (8), while the mean (SD) for HFrEF group was 34 (4).

### 3.1. Direct Cost

The total annual direct medical costs associated with HF during the period of the study was $3,528,839. The average annual direct medical cost per patient was $9563. The average annual direct medical costs in this study varied between patient age groups, ranging from $7456 in patients aged less than 40 years to $11,720 in patients aged over 60. In addition, the annual costs varied among the patients based on gender, HF class, and type of LVEF (Table 2). The average direct cost per patient varied according to their NYHA class and ranged from $3671 in class I to $16,447 in class IV (*p* = 0.002). In addition, we found the average direct cost per female patient ($9799) to be slightly higher than per male patient ($9431). Moreover, the average direct medical cost per HFrEF patient was $10,597, compared with $6480 per HFpEF patient (*p* = 0.01).

**Figure 1:**
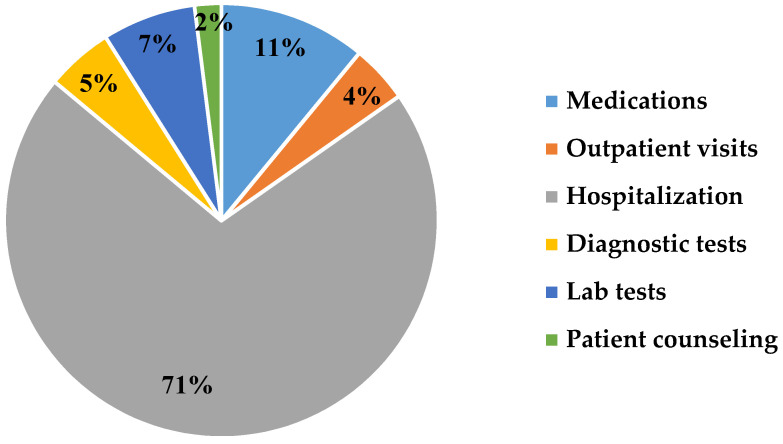
Major drivers of direct medical costs associated with heart failure in Saudi Arabia.

The study revealed that approximately 44% of patients were admitted or re-admitted for hospitalization, and the cost of managing them accounted for 71% of the total spending in direct costs (Figure 1). Hospitalization costs included medical expenses related to in-patient services consumed during the hospital stay. The average cost per hospitalized patient varied according to their NYHA class, ranging from $1364 in class I to $13,271 in class IV. Moreover, the average cost per hospitalized female patient was $7027, compared with $6702 per male patient. In addition, the average cost per HF patient classified as HFrEF was $7721, and $ 4128 per patient classified as HFpEF.

Costs related to the procedures adopted represented 54% of the hospitalization costs for all HF patients. Among the patients who required procurers, implantable cardioverter defibrillators (ICDs) represented 49% of the total number of procedures adopted during the period of study, while cardiac resynchronization therapy (CRT) accounted for 33%. Revascularization using percutaneous coronary intervention (PCI) accounted for 15% of the total number of procedures adopted, and coronary artery bypass graft (CABG) surgery accounted for 2%. The study also revealed that valve replacements and cardiac transplants accounted for only 1% of the total number of procedures adopted.

Our analysis found that the costs related to diagnostic tests were 7% of the total expenditure, while those related to laboratory tests were 5%. Among the different diagnostic tests adopted, electrocardiogram (ECG) accounted for 61%, echocardiogram accounted for 21%, and cardiac magnetic resonance imaging (CMRI) accounted for 8%. The average annual cost of diagnostic tests per patient was $715, while the average annual cost of laboratory tests was $522. Outpatient physician visits accounted for approximately 4% of the total expenditure in direct costs, with an average annual cost of $414 per patient. Education visits conducted by other healthcare professionals, such as pharmacists and nurses, accounted for 2% of the total expenditure in direct costs, while the estimated annual costs were $167 per HF patient. The cost of medications used for the standard care of patients with HF accounted for 11% of the total spending. The most commonly dispensed medications were beta-blockers (30%), diuretics (26%), angiotensin-converting enzyme inhibitors (ACEI) (15%), and statins (9%).

The study found that having comorbidities in addition to HF could result in a greater increase in the estimated annual direct cost per HF patient. The regression analysis revealed that each additional comorbidity was associated with an increase in the annual direct cost for an HF patient by $1397 (*p* = 0.02). On average, the direct medical cost for a HF patient also suffering from diabetes was $9973, from hypertension was $10,930, and from ischemic heart disease was $11,217.

### 3.2. Indirect Cost

A total of 100 patients in the study were actively working and were thus included in the analysis of indirect costs. This study found that the average number of hours lost per month due to absenteeism was 32 h per patient, and 44 h due to presenteeism per patient. Table 3 provides an estimation of the productivity loss due to HF in patients, based on the total number of hours lost due to absenteeism, presenteeism, and associated costs. Approximately 42% of the annual indirect costs were related to absenteeism, while 58% were related to presenteeism. The average annual indirect cost per patient resulting from absenteeism was $4628, compared with $6388 from presenteeism. The total indirect cost incurred during the period of the study for working HF patients was estimated at $1,100,160. As a result, the total cost for HF in this study, including direct and indirect costs, was estimated at $4,628,999.

## 4. Discussion

Heart failure is considered to be a major health problem with its prevalence in approximately 1% to 2% of the global population, causing a significant economic burden on all healthcare systems [15,33]. The estimation of the economic impact of HF on society is a great tool that provides valuable information about the use of health resources and the country’s expenditure on this disease. This will help decision makers in prioritizing healthcare policies, implementing interventions, and efficiently allocating the available health resources [29,34].

This study considers a broader perspective and accounts for the loss in productivity among affected patients. The study estimated the direct medical cost using a prevalence-based, bottom-up approach, which is known to provide an accurate estimation of the cost.

The findings of this study revealed that the economic burden of HF due to direct medical costs in Saudi Arabia is considerably high, estimated at $3,528,839 per year for the patients included in this study. This represents 76.3% of the total cost, while the annual average direct medical cost was $9563 per patient. This cost is less than the estimated annual average cost of $24,383 per patient, which ranges from $14,226 to $45,784 reported in a recent systemic review of 87 studies [23]. Furthermore, the annual cost for HF patients in Saudi Arabia was lower than the estimated cost reported in various countries, including $15,952 in Italy; $15,334 in Ireland; $24,873 in USA; and $25,532 in Germany. However, the reported cost in Saudi Arabia is higher than the annual costs for HF patients reported in South Korea, where costs are reported as ranging between $868 and $1560.5; $2343 in Nigeria; $4755 in Poland; $5044 in Sweden; $7053 in Greece; and $7792 in Spain [14,18]. This indicates that the large variations in cost reported from different countries may be due to the methodology used for estimating costs, treatment protocol, and the prices of healthcare resources. In addition, this highlights the importance of using local data to estimate the COI, as relying on international figures or other countries’ estimates can produce an inaccurate representation of the actual costs, which may impact on decision-making that may negatively affect patient care.

The findings of this study varied in comparison to previous studies conducted in Saudi Arabia. One of these earlier studies used a survey questionnaire where clinical experts reported the direct cost of HF to be $8137 per patient per year [25]. This study may have underestimated the actual direct cost of HF, as it is lower than our observation ($9563). This could be explained through a difference in the methodology used in the two studies. Our study used a more comprehensive approach that is commonly used in COI studies, relying on actual patient-level data, resource utilization, and cost data, rather than on expert opinions.

Moreover, another study conducted in a single center in the city of Mekkah focused on the hospitalization costs associated with HFrEF patients and estimated that the costs ranged from $909, for a one-day hospitalization, to $7999 per single hospitalization. In addition, the annual hospitalization cost was $12,311, compared with $7721 per HFrEF patient in our study [26]. Such a large variation may be because there were no standard treatment guidelines or protocols for patients with HF. However, in response to these variations in clinical practice, the Saudi Hearth Association (SHA) has developed guidelines for the diagnosis and management of HF in Saudi Arabia [35].

Our study demonstrated existing differences in costs between gender and across different HF classes. The annual direct cost for female patients was found to be $9799 per patient, which is higher when compared to $9431 per male patient but is considered to be an insignificant variation. This result is in line with a previous study conducted in Sweden [36].

Furthermore, the direct costs of HF management increased significantly as the disease progressed to advanced classes. The cost ranged between $3671 in NYHA class I to $16,447 in class IV. For a hospitalized patient, it varied between $1364 in class I and $13,271 in class IV. This significant increase in costs could be explained by the increased demand for treatment and higher consumption of healthcare resources by patients with advanced classes. Our finding is in line with previous studies conducted in Spain and Poland that reported a significant difference between patients in the NYHA class II and patients in classes III or IV [37,38]. This highlights the importance of early diagnosis and treatment of HF in order to prevent the condition from progressing to a more advanced class or severe case, which is more symptomatic and costly.

In this study, the cost was $6480 per HFpEF patient and $10,597 per HFrEF patient. It should be noted that the majority of patients were classified as HFpEF, which is the dominant form of HF. Prior studies have demonstrated that the total annual cost of HFrEF was higher than that of HFpEF (€13,011 and €12,206), but that the long-term costs of HFpEF were found to be higher mainly because of re-hospitalization [39].

Patients with comorbidities, such as diabetes, hypertension, and ischemic heart disease, will have added complexity in the treatment and require additional health resources that increase the cost of treatment. This is because each additional comorbidity is estimated to increase the annual treatment cost by $1397. These conditions should be considered, as many patients with HF face other comorbidities. Similar findings were reported in one study conducted in Spain that found a consistent increase in the annual cost per HF patient among patients with a high number of existing comorbidities, reaching up to $19,865 in patients with more than 9 comorbidities [40].

Hospitalization, including for procedures, accounted for a high percentage of the total expenditure on direct costs in the case of HF patients. Annual hospitalization costs were approximately $6819 per HF patient, which accounts for 71% of the total direct cost per patient. This indicates the importance of planning an effective prevention strategy in order to minimize the hospitalization rate and decrease the total expenditure on HF patients. In countries such as the UK, hospital admissions for HF patients account for 73% of the total spending, which is quite similar to our estimate [20]. Despite the difference in cost components reported across different studies, the hospitalization costs were the main driver of the total cost of treatment for HF patients in many countries [22].

In this study, medication costs account for 11% of the total cost, which is higher than in countries such as Spain (7%) and lower than in countries such as Nigeria (15%) [40,41]. This variation in cost components is primarily due to the different medication, treatment guidelines, and drug prices available in each country.

In this study, the indirect cost was estimated based on a human capital approach. Unlike prior local studies, we estimated the impact of the loss in productivity in terms of the presenteeism and absenteeism of HF patients, with the aim to fill the research gap around indirect costs. The indirect cost is often missing from studies, mainly because of the unavailability of data and difficulty in its calculation. Estimating the indirect cost, along with the direct cost, in this study provided a broad perspective of the total cost of treatment for HF patients in Saudi Arabia. As HF can have a negative impact on patients’ ability to work, we found that an average patient lost approximately 32 h per month due to absenteeism and 44 h per month due to presenteeism. The estimated annual indirect cost per patient resulting from absenteeism was $4628 and presenteeism was $6388, with a total of $1,100,160 for 100 patients, which represented 23.7% of the total HF cost in the study.

The indirect cost was significantly high in many countries; for example, the indirect cost in the form of informal care ranged from 59–69.8% in Spain and represented approximately 44% of the total cost in Nigeria [38,42]. Moreover, the total indirect cost, which accounted for mortality cost, home productivity, and absenteeism, was reported as $10.6 billion in the US (0.071% of GDP) [42]. Similarly, in Poland, the cost was estimated at €871.9 million in 2012, and projected to reach €945.3 million in 2015 [43]. These findings were higher than those reported in our study, largely because our estimates for indirect cost results were limited to 100 HF patients. However, our findings for indirect costs could add an important insight about the influence of indirect costs that are attributable to HF in Saudi Arabia, given that the age of diagnosis of HF in Saudi patients is considerably younger than global estimates [12]. This might affect the patient’s productivity in the labor market, which, in turn, results in a substantial increase in the indirect cost on a national level.

## 5. Strength and Limitations

This study aimed to estimate the cost of HF treatment using real world data and taking into consideration broader perspectives and standard COI methodology. However, the study has some limitations. In the estimation of direct costs, the study focused on direct medical costs and did not include direct non-medical costs, such as that of transportation and housing for patients visiting from outside Riyadh. Although the study used a representative sample of HF patients from two large public healthcare institutions in Riyadh, it did not account for those in other large institutions at a national level and from the private sector. The study also did not account for HF patients with midrange EF (HFmrEF), due to limited data available for this group. Future studies may consider focusing on a nationally representative HF sample and estimate other costs, such as direct non-medical costs.

## 6. Conclusions

Heart failure, which is considered to be a major health problem in Saudi Arabia, imposes a significant economic burden on its healthcare system and society. The findings of this study may help in providing valuable information about the country’s use of health resources and expenditure incurred on this disease. This will help decision makers in prioritizing healthcare policies, implementing interventions, and efficiently allocating the available health resources.

## Figures and Tables

**Table 1:** Patient’s demographics and disease characteristics.

Variable	Count (*N* = 369)	Frequency (%)
Gender		
Male	238	64%
Female	131	36%
Age		
Less than 40	73	20%
40–60	176	48%
More than 60	119	32%
Smoking		
No	282	76%
Yes	87	24%
Disease duration		
Less than 1 year	64	17%
1–5 years	168	46%
5–10 years	107	29%
More than 10 years	30	8%
HF NYHA class		
I	98	29%
II	120	38%
III	84	23%
IV	67	10%
HF by LVEF		
HFrEF	93	25%
HFpEF	276	75%
Comorbidities		
Diabetes	189	51%
Hypertension	164	44%
Ischemic heart disease	109	29%
Dyslipidemia	52	14%

Abbreviations: HF, Heart failure; NYHA, New York Heart Association; LVEF, Left ventricular ejection fraction; HFrEF, Heart failure reduced ejection fraction; HFpEF, Heart failure preserved ejection fraction.

**Table 2:** The average annual direct medical costs by components per HF patient.

Variable	Medication	Visits	Hospitalization	Diagnostics	Lab Tests	Education	**Cost/Patient**
NYH class							
I	$819 (76)	$330 (23)	$1364 (763)	$424 (44)	$569 (67)	$165 (10)	$3671 (697)
II	$869 (106)	$437 (28)	$8691 (2455)	$561 (56)	$818 (72)	$168 (8)	$11,544 (1970)
III	$1057 (148)	$455 (44)	$9289 (2619)	$576 (76)	$688 (87)	$169 (10)	$12,236 (2242)
IV	$1166 (226)	$482 (61)	$13,271 (4475)	$543 (123)	$817 (145)	$168 (17)	$16,447 (3668)
Gender							
Female	$866 (97)	$405 (28)	$7027 (2293)	$538 (56)	$795 (69)	$168 (10)	$9799 (1738)
Male	$958 (73)	$419 (25)	$6702 (1690)	$514 (69)	$671 (85)	$167 (9)	$9431 (1271)
HF type LVEF							
HFpEF	$797 (106)	$328 (55)	$4128 (1660)	$460 (62)	$611 (71)	$156 (11)	$6480 (1459)
HFrEF	$969 (69)	$443 (68)	$7721 (1596)	$543 (38)	$750 (50)	$171 (6)	$10,597 (1268)

Abbreviations: HF, Heart failure; NYHA, New York Heart Association; LVEF, Left ventricular ejection fraction; HFrEF, Heart failure reduced ejection fraction; HFpEF, Heart failure preserved ejection fraction.

**Table 3:** The estimated indirect cost associated with heart failure.

Variable	Absenteeism	Presenteeism
Number of hours missed per year	384 (89)	528 (47)
Estimated indirect cost/patient	$4628 (1757)	$6388 (1013)
Estimated indirect cost as % of GDP per capita	20%	27%

Notes: The Saudi GDP per capita used for the analysis was $23,139.80 and, accordingly, the average wage per hour was $12. The estimated hours lost due to absenteeism or presenteeism was based on the assumption of 40 working hours per week. Hours and costs are presented as mean (SD).

## Data Availability

Data sharing not applicable.
